# Efficacy and safety of lenvatinib in patients with recurrent hepatocellular carcinoma after liver transplantation

**DOI:** 10.1002/cam4.5123

**Published:** 2022-08-05

**Authors:** Kyunghye Bang, Andrea Casadei‐Gardini, Changhoon Yoo, Massimo Iavarone, Min‐Hee Ryu, Sook Ryun Park, Hyung‐Don Kim, Young‐In Yoon, Dong‐Hwan Jung, Gil‐Chun Park, Chul‐Soo Ahn, Deok‐Bog Moon, Shin Hwang, Ki‐Hun Kim, Gi‐Won Song, Chiara Mazzarelli, Eleonora Alimenti, Stephen L. Chan, Massimo De Giorgio, Baek‐Yeol Ryoo, Sung‐Gyu Lee

**Affiliations:** ^1^ Department of Oncology, Asan Medical Center University of Ulsan College of Medicine Seoul Republic of Korea; ^2^ Department of Medical Oncology Vita‐Salute San Raffaele University, IRCCS San Raffaele Scientific Institute Milan Italy; ^3^ Division of Gastroenterology and Hepatology Foundation IRCCS Ca' Granda Ospedale Maggiore Policlinico Milan Italy; ^4^ Division of Hepatobiliary Surgery and Liver Transplantation Department of Surgery, Asan Medical Center, University of Ulsan College of Medicine Seoul Republic of Korea; ^5^ Hepatology and Gastro‐Enterology Unit ASST Ospedale Niguarda Milan Italy; ^6^ State Key Laboratory of Translational Oncology Department of Clinical Oncology, Sir YK Pao Centre for Cancer, The Chinese University of Hong Kong Hong Kong; ^7^ Department of Gastroenterology Hepatology and Liver Transplantation, Papa Giovanni XXIII Hospital Bergamo Italy; ^8^ Division of Hemato‐Oncology, Department of Internal Medicine Chung‐Ang University Gwangmyeong Hospital Gwangmyeong Republic of Korea

**Keywords:** Albumin–bilirubin grade, chemotherapy, hepatocellular carcinoma, lenvatinib, liver transplantation

## Abstract

**Introduction:**

Lenvatinib is approved for the treatment of patients with metastatic or recurrent hepatocellular carcinoma (HCC); however, clinical outcomes of lenvatinib therapy in patients with post‐liver transplantation (LT) HCC recurrence remain unclear. We investigated the efficacy and safety of lenvatinib in patients with post‐LT HCC recurrence.

**Methods:**

This multinational, multicenter, retrospective study included 45 patients with recurrent HCC after LT who received lenvatinib at six institutions in three countries (Korea, Italy, and Hong Kong) from June 2017 to October 2021.

**Results:**

At the time of lenvatinib initiation, 95.6% (*n* = 43) of patients had Child–Pugh A status, and 35 (77.8%) and 10 (22.2%) participants were classified as having albumin–bilirubin (ALBI) grades 1 and 2, respectively. The objective response rate was 20.0%. With a median follow‐up duration of 12.9 months (95% confidence interval [CI]: 11.2–14.7), the median progression‐free survival and overall survival (OS) were 7.6 (95% CI: 5.3–9.8) months, and 14.5 (95% CI: 0.8–28.2) months, respectively. Patients with ALBI grade 1 showed significantly better OS (52.3 months, [95% CI: not assessable]) than patients with ALBI grade 2 (11.1 months [95% CI: 0.0–30.4 months], *p* = 0.003). The most common adverse events were hypertension (*n* = 25, 55.6%), fatigue (*n* = 17, 37.8%), and anorexia (*n* = 14, 31.1%).

**Conclusion:**

Lenvatinib showed consistent efficacy and toxicity profiles in patients with post‐LT HCC recurrence that were comparable to those reported from previous studies among non‐LT HCC patients. The baseline ALBI grade correlated with better OS in post‐LT lenvatinib‐treated patients.

## Graphical abstract

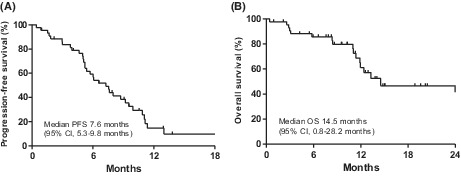

## 1 INTRODUCTION

Worldwide, hepatocellular carcinoma (HCC) is the sixth most common cancer and the third leading cause of cancer‐related mortality.^1^ HCC is an extremely heterogenous disease, and the selection of the treatment strategy may vary, based on tumor burden, the degree of underlying liver cirrhosis, and the patient's performance status. Surgical resection, liver transplantation (LT), and locoregional therapies, such as radiofrequency ablation (RFA), are all potentially curative therapeutic modalities for patients with early‐stage HCC.^2^ LT offers the possibility of curing both the tumor and underlying liver diseases, including cirrhosis. However, post‐LT HCC recurrence has been reported in 10%–18% of patients, and the median interval from LT to HCC recurrence is 12–13 months.^3^, ^4^, ^5^ Based on the recurrence pattern in patients with post‐LT HCC recurrence, local therapies such as RFA, transarterial chemoembolization (TACE), and surgical resection may be considered. Systemic therapy should be considered in patients with extrahepatic metastasis or for those whose tumors may be refractory to local therapy.^5^

For more than a decade, sorafenib – an oral multikinase inhibitor (MKI) – was the only systemic drug that was available for patients with post‐LT HCC recurrence. Moreover, most of the small retrospective studies that evaluated the outcomes of sorafenib treatment have shown variable clinical outcomes, with 1‐year overall survival (OS) rates of 18%–90%.^6^, ^7^

Lenvatinib is an MKI for vascular endothelial growth factor (VEGF) receptors 1–3, fibroblast growth factor receptors 1–4, PDGFRα, RET, and KIT. In a phase 3, multicenter, randomized trial (REFLECT), as compared with sorafenib, lenvatinib demonstrated non‐inferior OS and statistically significant improvements in progression‐free survival (PFS), time to progression, and the objective response rate (ORR).^8^ Based on the abovementioned data, lenvatinib was approved as a first‐line standard therapy for unresectable or metastatic HCC. However, patients with prior LT were excluded from prospective studies of lenvatinib.^8^, ^9^, ^10^ As only a limited number of studies, with most including <15 patients, have been reported,^11^, ^12^, ^13^, ^14^ further investigation is required to assess the efficacy and safety of lenvatinib in patients with post‐LT HCC recurrence. Therefore, we conducted a multinational, multicenter, retrospective study of lenvatinib in patients with post‐LT HCC recurrence.

## 2 MATERIALS AND METHODS

### 2.1 Patients

Patients who were treated with lenvatinib for post‐LT HCC recurrence from June 2017 to October 2021 were identified from six centers in three countries (Korea, Italy, and Hong Kong). Clinical and laboratory data were retrospectively obtained by reviewing the electronic medical records. This study was approved by the institutional review board of each participating center and was performed in accordance with the ethical standards specified in the institutional research guidelines and the principles evinced in the Declaration of Helsinki.

### 2.2 Treatment and evaluation

Lenvatinib was administered at the standard dosage for patients with advanced HCC, based on the dosage that was described in the REFLECT trial (12 or 8 mg/day for patients with body weight ≥60 or <60 kg, respectively).^8^ Dose modification (to 8 or 4 mg/day) at the initiation or during the lenvatinib treatment course was allowed at the discretion of the attending physicians.

Patients underwent computed tomography or magnetic resonance imaging every 6–8 weeks for the assessment of the tumor status. The investigators from various institutions graded the tumor response according to the Response Evaluation Criteria in Solid Tumors version 1.1 (RECIST v1.1). All treatment‐related adverse events (AEs) were graded according to the National Cancer Institute Common Terminology Criteria for Adverse Events version 5.0.

### 2.3 Statistical analysis

The ORR and disease control rate (DCR) were evaluated according to the RECIST v1.1. PFS was defined as the interval from the initiation of lenvatinib to the date of disease progression or death, whichever occurred first. OS was defined as the time from the initiation of lenvatinib to death from any cause. The time to response (TTR) was defined as the interval between the initiation of lenvatinib and the best response. Survival outcomes were estimated using the Kaplan–Meier method. A two‐sided *p*‐value <0.05 was considered statistically significant. All statistical analyses were performed using the Statistical Package for the Social Sciences version 23.0 (IBM).

## 3 RESULTS

### 3.1 Patient characteristics

A total of 45 patients who received lenvatinib for post‐LT recurrent HCC were included in this analysis. The baseline patient characteristics are summarized in Table 1. The median age was 59 years (range, 20–87 years), and the majority of the study population (*n* = 43, 95.6%) was male. All patients had an Eastern Cooperative Oncology Group performance status of 0 or 1. The most common HCC etiology was hepatitis B (*n* = 25, 55.6%), followed by hepatitis C (*n* = 11, 24.4%) and alcohol consumption (*n* = 4, 8.9%).

**TABLE 1:** Baseline characteristics of the patients

Variables	Total *N* = 45 *n* (%) or median (range)
Sex	
Male	43 (95.6%)
Female	2 (4.4%)
Age, years, median (range)	59 (20–87)
Ethnicity	
East Asian	24 (53.3%)
Caucasian	21 (46.7%)
ECOG performance status	
0	19 (42.2%)
1	26 (57.8%)
Etiology	
Hepatitis B	25 (55.6%)
Hepatitis C	11 (24.4%)
Alcohol	4 (8.9%)
Others	5 (11.1%)
Child–Pugh score	
A	43 (95.6%)
B	2 (4.4%)
ALBI grade	
1	35 (77.8%)
2	10 (22.2%)
Site of recurrence or metastasis	
Liver	29 (64.4%)
Lung	24 (53.3%)
Peritoneum	11 (24.4%)
Bone	9 (20.0%)
Lymph node	8 (17.8%)
AFP, U/ml, median (range)	37 (1–373,072)
<400	31 (68.9%)
≥400	14 (31.1%)
Reason for liver transplantation	
Hepatocellular carcinoma	45 (100.0%)
Liver transplantation type	
LDLT	21 (46.7%)
DDLT	24 (53.3%)
Immunosuppressants	
Tacrolimus	41 (91.1%)
Everolimus	34 (75.6%)
Mycophenolate mofetil	5 (11.1%)
Liver transplantation–lenvatinib‐therapy‐initiation interval, months, median (range)	28.1 (4.2–231.9)
BCLC stage	
B	4 (8.9%)
C	41 (91.1%)
Treatment line of lenvatinib	
First	42 (93.3%)
Second	3 (6.7%)

Abbreviations: AFP, alpha‐fetoprotein; ALBI, albumin–bilirubin; BCLC, Barcelona Clinic Liver Cancer; DDLT, deceased donor liver transplantation; ECOG, Eastern Cooperative Oncology Group; LDLT, living donor liver transplantation.

All patients had previously received LT for the management of HCC, and approximately half (*n* = 21, 46.7%) had received living‐donor liver transplantations. Most of the patients received a combination of immunosuppressants, including tacrolimus (TAC; *n* = 41, 91.1%), everolimus (EVE; *n* = 34, 75.6%), or mycophenolate mofetil (MMF; *n* = 5, 11.1%). The distribution of immunosuppressant use was as follows: TAC‐only (*n* = 6); EVE‐only (*n* = 2); MMF‐only (*n* = 1); TAC + EVE (*n* = 31); TAC + MMF (*n* = 2); and TAC + EVE + MMF (*n* = 1). The median time to recurrence of HCC after LT was 22.4 months (95% confidence interval [CI]: 1.1–207.0 months). Prior to systemic therapy, TACE (*n* = 17, 37.8%) was the most commonly used therapy for post‐LT HCC recurrence, followed by surgical resection (*n* = 15, 33.3%) and radiotherapy (*n* = 8, 17.8%).

At the time of lenvatinib treatment, most patients had Barcelona Clinic Liver Cancer stage C disease (*n* = 41, 91.1%) and the most common sites of recurrence were the liver (*n* = 29, 64.4%) and lungs (*n* = 24, 53.3%), followed by the peritoneum (*n* = 11, 24.4%) and bone (*n* = 9, 20.0%). Most patients (*n* = 43, 95.6%) had a Child–Pugh A liver function, and 35 (77.8%) and 10 (22.2%) patients had albumin–bilirubin (ALBI) grades 1 and 2, respectively.

Most patients (*n* = 42, 93.3%) received lenvatinib as the first‐line therapy. Three patients (6.7%) received lenvatinib as a second‐line therapy after progression on first‐line sorafenib. The median interval between LT and the initiation of lenvatinib therapy was 28.1 months (range, 4.2–231.9 months), and the starting dose of lenvatinib was 12 mg/day for 29 patients (64.4%) and 8 mg/day for the remaining 15 patients (33.3%). Seven patients with a body weight > 60 kg received a reduced starting dose of lenvatinib, and the commonest reasons for the dose down‐titration were decreased renal (*n* = 2) and hepatic (*n* = 1) function and tumor bleeding (*n* = 1). At the data cutoff point (December 9, 2021), 13 (28.9%) patients were receiving lenvatinib treatment (1.0+ to 20.0+ months), and the median duration of lenvatinib treatment was 6.6 months (range, 0.1–20.0 months). Tumor progression was the commonest reason for the discontinuation of lenvatinib (28 of 35, 80.0%). Seventeen patients died, and the cause of death for all of these patients was HCC progression.

### 3.2 Efficacy of lenvatinib

**FIGURE 1:**
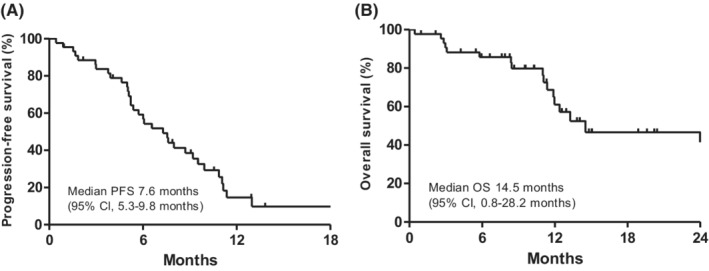
Kaplan–Meier curves of (A) PFS and (B) OS following lenvatinib therapy in patients with recurrent hepatocellular carcinoma after liver transplantation. CI, confidence interval; OS, overall survival; PFS, progression‐free survival.

Efficacy outcomes in lenvatinib‐treated patients with recurrent HCC after LT are summarized in Table 2. According to the RECIST v1.1, partial response (PR), stable disease, and progressive disease were graded in 9 (20.0%), 31 (68.9%), and 3 (6.7%) patients, respectively. None of the patients achieved a complete response (CR). The ORR was 20.0% and the DCR was 88.9%. In patients who achieved partial response, the median TTR was 2.4 months (95% CI: 1.5–7.4 months). With a median follow‐up duration of 12.9 months (95% CI: 11.2–14.7 months), median PFS and OS were 7.6 months (95% CI: 5.3–9.8 months) and 14.5 months (95% CI: 0.8–28.2 months), respectively (Figure 1). The 6‐month PFS and OS rates were 60.1% and 86.0%, respectively.

**TABLE 2:** Efficacy of lenvatinib in patients with recurrent hepatocellular carcinoma after liver transplantation

Variables	Lenvatinib (*N* = 45)
Best response	
CR	0 (0.0%)
PR	9 (20.0%)
SD	31 (68.9%)
PD	3 (6.7%)
Not evaluable	2 (4.4%)
Overall response rate	20.0%
Disease control rate	88.9%
Median TTR, months (range)	2.4 (1.5–7.4)
Median PFS, months (95% CI)	7.6 (5.3–9.8)
6‐month PFS rate	60.1%
Median OS, months (95% CI)	14.5 (0.8–28.2)
6‐month OS rate	86.0%

Abbreviations: CI, confidence interval; CR, complete response; OS, overall survival; PD, progressive disease; PFS, progression‐free survival; PR, partial response; SD, stable disease; TTR, time to response.

**FIGURE 2:**
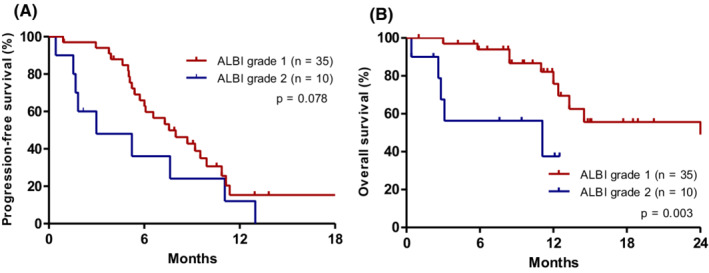
Kaplan–Meier curves of (A) PFS and (B) OS according to the albumin–bilirubin (ALBI) grade at the time of lenvatinib initiation.

When stratified by the ALBI grade (grade 1 vs. grade 2), patients with ALBI grade 1 had a numerically higher median PFS (8.0 months [95% CI: 5.2–10.8 months]) than those with ALBI grade 2 (3.0 months [95% CI: 0.0–7.5 months]; *p* = 0.078; Figure 2A). Moreover, patients with ALBI grade 1 had significantly better OS (52.3 months [95% CI: not assessable]) compared to patients with ALBI grade 2 status (11.1 months [95% CI: 0.0–30.4 months]; *p* = 0.003; Figure 2B).

The PFS or OS did not differ significantly by the immunosuppressant regimens at the time of lenvatinib treatment initiation (EVE‐containing regimens vs. others, *p* = 0.384 [PFS] and *p* = 0.480 [OS]; Figure S1). The time to HCC recurrence after LT (< median [22.4 months] vs. ≥ median) was not significantly associated with the median PFS (7.6 months [95% CI: 4.5–10.7] vs. 7.3 months [95% CI: 4.3–10.3]; *p* = 0.826) or OS (52.6 months [95% CI: not assessable] vs. 13.3 months [95% CI: 10.1–16.5]; *p* = 0.282; Figure S2). In addition, the recurrence pattern at the time of lenvatinib initiation (only intrahepatic recurrence [*n* = 10] vs. extrahepatic metastasis [*n* = 35]) did not show any relationship with median PFS (5.4 months [95% CI: 4.5–6.3] vs. 7.6 months [95% CI: 5.8–9.5]; *p* = 0.089) or OS (11.4 months [95% CI: 10.9–not reached] vs. 52.3 months [95% CI: not assessable]; *p* = 0.204; Figure S3).

### 3.3 Subsequent therapy

Among the 31 patients with HCC progression on lenvatinib therapy, 22 (71.0%) received subsequent systemic therapy. Sorafenib was the most commonly used agent (*n* = 19, 86.4%), followed by cabozantinib (*n* = 2, 9.1%) and regorafenib (*n* = 1, 4.5%). None of the patients underwent re‐transplantation for recurrent HCC after commencing lenvatinib treatment.

### 3.4 Safety profiles

Lenvatinib therapy was interrupted or the dose was reduced in 22 (48.9%) patients. The most common cause of dose modification was fatigue (7/22, 31.8%), followed by hypertension (4/22, 18.2%), and proteinuria (2/22, 9.1%). AEs that occurred in >5% of patients are listed in Table 3. The majority (*n* = 44, 97.8%) of patients experienced an AE. Hypertension (*n* = 25, 55.6%) was the most frequent AE, followed by fatigue (*n* = 17, 37.8%), and liver enzyme elevation (*n* = 17, 37.8%). The most common grade 3–4 AEs were hypertension (*n* = 9, 20.0%) and neutropenia (*n* = 4, 8.9%). Four (8.9%) patients discontinued lenvatinib due to AEs (*n* = 2, grade 3 hypertension; *n* = 1, grade 3 proteinuria; *n* = 1, grade 3 fatigue).

**TABLE 3:** Adverse events during lenvatinib treatment

	Adverse Events (Total *N* = 45)				
	Any grade *n* (%)	Grade 1 *n* (%)	Grade 2 *n* (%)	Grade 3 *n* (%)	Grade 4 *n* (%)
All	44 (97.8%)	36 (80.0%)	27 (60.0%)	16 (25.6%)	0 (0.0%)
Neutropenia	7 (15.6%)	1 (2.2%)	2 (4.4%)	4 (8.9%)	0 (0.0%)
Anemia	7 (15.6%)	5 (11.1%)	2 (4.4%)	0 (0.0%)	0 (0.0%)
Thrombocytopenia	8 (17.8%)	7 (15.6%)	1 (2.2%)	0 (0.0%)	0 (0.0%)
Elevated AST/ALT	17 (37.8%)	17 (37.8%)	3 (6.7%)	0 (0.0%)	0 (0.0%)
Elevated bilirubin	4 (8.9%)	4 (8.9%)	0 (0.0%)	0 (0.0%)	0 (0.0%)
Hypothyroidism	10 (22.2%)	7 (15.6%)	3 (6.7%)	0 (0.0%)	0 (0.0%)
Hypertension	25 (55.6%)	9 (20.0%)	7 (15.6%)	9 (20.0%)	0 (0.0%)
Proteinuria	10 (22.2%)	3 (6.7%)	5 (11.1%)	2 (4.4%)	0 (0.0%)
Fatigue	17 (37.8%)	8 (17.8%)	5 (11.1%)	4 (8.9%)	0 (0.0%)
Anorexia	14 (31.1%)	9 (20.0%)	4 (8.9%)	1 (2.2%)	0 (0.0%)
Diarrhea	12 (26.7%)	7 (15.6%)	5 (11.1%)	0 (0.0%)	0 (0.0%)
Hand–foot syndrome	7 (15.6%)	6 (13.3%)	1 (2.2%)	0 (0.0%)	0 (0.0%)
Oral mucositis	6 (13.3%)	6 (13.3%)	0 (0.0%)	0 (0.0%)	0 (0.0%)

Abbreviations: ALT, alanine aminotransferase; AST, aspartate aminotransferase.

## 4 DISCUSSION

This multinational, multicenter, retrospective analysis of patients with post‐LT HCC recurrence demonstrated that lenvatinib showed an ORR of 20.0% as well as median PFS and OS of 7.6 and 14.5 months, respectively. Our findings on the efficacy and safety of lenvatinib in patients with LT are comparable to those of the pivotal phase 3 REFLECT trial (ORR, based on RECIST v1.1, 18.8%; median PFS 7.4 months; and median OS 13.6 months), which excluded patients with prior LT,^8^ and of previous real‐world studies.^15^, ^16^, ^17^, ^18^ Our findings validate the clinical relevance of lenvatinib therapy in patients with recurrent HCC following LT.

Although immune checkpoint inhibitors are now considered key treatment components in the management of patients with advanced HCC,^19^, ^20^, ^21^ patients with prior LT may not benefit from the abovementioned therapy because of the risk of allograft rejection.^22^ Therefore, targeted agents, mainly MKIs, should be the mainstay for managing patients with unresectable or metastatic HCC following LT. However, as patients with prior LT have been excluded from previous prospective randomized trials of currently approved agents for unresectable or metastatic HCC, an optimal strategy for systemic therapy in patients with recurrent HCC following LT has not been established.

As sorafenib is the only systemic therapy that has been approved for unresectable or metastatic HCC, most studies of systemic therapy in patients with recurrent HCC after LT have included sorafenib^23^, ^24^, ^25^, ^26^, ^27^; however, most of the abovementioned studies had small sample sizes (*n* = 5–50). In a previous meta‐analysis of sorafenib for patients with recurrent HCC after LT, the median OS was 10.5 months (range, 5–21.3 months) and the median percentage of patients who achieved CR and PR was 0% (range, 0%–11.7%) and 4.8% (range, 0%–26.7%), respectively.^6^ Our findings suggest that lenvatinib may have better efficacy outcomes than sorafenib, as the ORR was 20% and the median OS was 14.5 months in our study, although a direct comparison between our results for lenvatinib and those that were reported for sorafenib in previous studies was not feasible. Further studies are necessary to define the optimal first‐line therapy in patients with post‐LT HCC recurrence.

Previous studies have shown that baseline liver function, as classified by the ALBI grade, is associated with the efficacy of lenvatinib in non‐LT patients with unresectable or metastatic HCC.^28^ Consistent with these results, a lower ALBI grade was significantly associated with better OS and was marginally related with better PFS with lenvatinib therapy in our study population. As multiple targeted agents become available for the management of HCC, it may be important to facilitate timely initiation of these drugs prior to the deterioration of liver function, even in patients with a prior LT, because repeated TACE may induce deterioration of liver function.^29^, ^30^ Nonetheless, immunosuppressants were not associated with the efficacy of lenvatinib against recurrent HCC following LT, although a previous study for combination of sorafenib and mTOR inhibitors, including EVE, showed favorable survival outcomes.^27^ There were four cases in which the TAC dose was adjusted. The TAC dose was increased only in one participant due to the decreased FK level during lenvatinib therapy. Of the other three patients who had their TAC dose reduced during lenvatinib treatment, one patient reduced the TAC dose and discontinued EVE due to proteinuria. However, none of the patients had graft rejection. Large multicenter studies are needed to define the relevant immunosuppressive regimens in combination with anticancer agents for post‐LT HCC recurrence.

The safety profiles of lenvatinib in patients with prior LT were consistent with the results of the REFLECT trial and other real‐world studies of lenvatinib in non‐LT HCC patients.^8^, ^13^, ^15^, ^31^ Although multiple immunosuppressants were used simultaneously with lenvatinib, no new safety signal was observed for lenvatinib in our study.

It is relevant to mention the limitations of our study, including the retrospective design, which is subject to unintentional biases. As almost all of the participants (95.6%) were male, this imbalance in the sex distribution may limit the generalizability of the results. Although the current analysis was based on the largest sample size for patients with prior LT, multivariate analysis to define the prognostic factors could not be performed because of insufficient statistical power. However, we included a diverse range of ethnic groups from multiple countries under various patterns of clinical practice.

## 5 CONCLUSIONS

In conclusion, lenvatinib showed consistent efficacy and toxicity patterns in patients with post‐LT HCC recurrence compared to those identified in the pivotal phase 3 REFLECT trial, which excluded patients with prior LT. Better liver function (ALBI grade 1) at the time of lenvatinib initiation correlated with better survival outcomes.

## AUTHOR CONTRIBUTIONS

Study concept: CY; Study design: CY, ACG, MI, S‐GL; Data acquisition: All authors; Data analysis and interpretation: All authors; Statistical analysis: KB, CY; Manuscript preparation: KB, ACG, CY, MI, S‐GL; Manuscript editing: All authors; Manuscript review and approval: All authors.

## FUNDING INFORMATION

This research received no specific grant from any public, commercial, or not‐for‐profit funding agency.

## CONFLICT OF INTERESTS

CY received grants from Bayer, ONO Pharmaceuticals, and AstraZeneca and has consultancy and advisory roles with Bayer, Eisai, Ipsen, Merck Sharp & Dohme (MSD), AstraZeneca, and Bristol Myers Squibb (BMS). ACG received grants from AstraZeneca and has consultancy and advisory roles with Bayer, Eisai, Ipsen, MSD, GlaxoSmithKline, and AstraZeneca. MI has speaking/teaching and consultancy roles and is on the advisory board for Bayer, Gilead Sciences, BMS, Janssen, Ipsen, MSD, BTG‐Boston Scientific, AbbVie, Guerbet, Eisai, and Roche. The other authors have no conflicts of interest to declare. CM received travel grant from Ipsen, and has advisory role with MSD and Ipsen.

## STUDY APPROVAL STATEMENT

The study protocol was approved by the Institutional Review Boards of Asan Medical Center (approval number 2020–1214), IRCCS San Raffaele Scientific Institute (DSAN854‐A‐OS/5), Sir YK Pao Centre for Cancer (2019.219), Foundation IRCCS Ca′ Granda Ospedale Maggiore Policlinico, ASST Ospedale Niguarda, and Papa Giovanni XXIII Hospital (480_2018bis). This study was performed in accordance with the ethical standards of the institutional research body and the principles evinced in the Declaration of Helsinki.

## CONSENT TO PARTICIPATE STATEMENT

The need for informed consent in this study was waived in consideration of the retrospective nature of this analysis.

## Supporting information


Appendix S1

## Data Availability

All of the data that were generated or analyzed during this study are included in this article and its supplementary material files. Further enquiries can be directed to the corresponding author.
