# Supplementary material for: Efficacy and safety of lenvatinib in patients with recurrent hepatocellular carcinoma after liver transplantation
Source: Cancer Med. 2022 Aug 5;12(3):2572–9. doi: 10.1002/cam4.5123 (PMC9939097; doi:10.1002/cam4.5123)
Supplement: Supplementary file 1 — Appendix S1 [file CAM4-12-2572-s001.docx]

**Supplementary Fig. 1. Kaplan–Meier curves of (a) progression-free survival and (b) overall survival according to immunosuppressant regimens.**

The median progression-free survival was 7.3 months (95% confidence interval [CI], 5.2–9.4) for EVE-containing regimens and 11.1 months (95% CI, 2.4–19.8) for other regimens. The median overall survival time was 13.3 months (95% CI, 9.6–17.0) in the groups receiving the EVE-containing regimens, but this target outcome was not reached for patients receiving other regimens. EVE, everolimus.

(a)


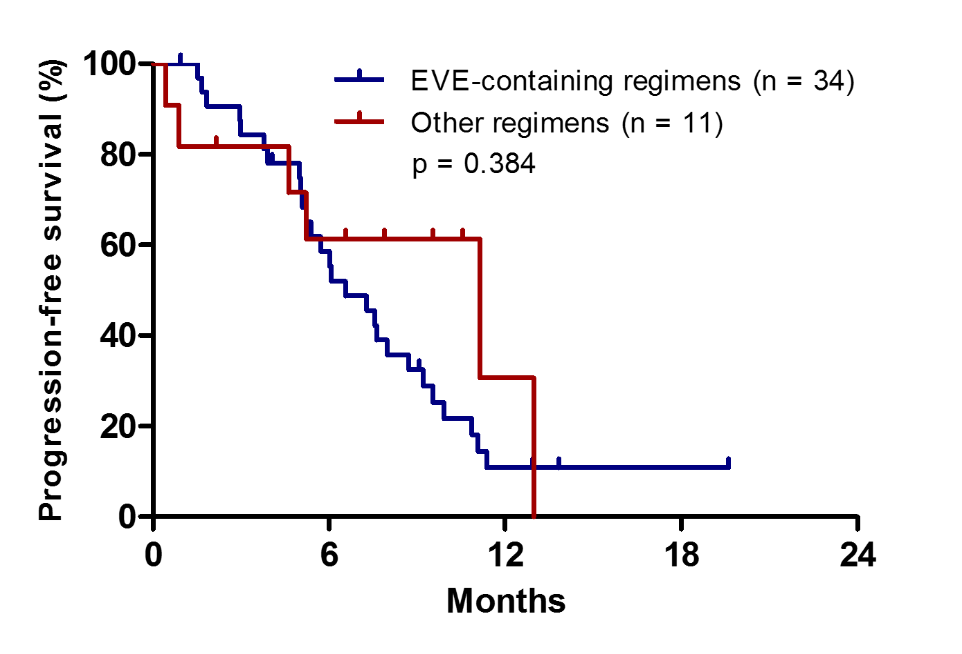


(b)

**Supplementary Fig. 2. Kaplan–Meier curves of (a) progression-free survival and (b) overall survival according to the median time to recurrence after liver transplantation (LT).**

The median time to recurrence after LT was 22.4 months.

(a)

(b)

**Supplementary Fig. 3. Kaplan–Meier curves of (a) progression-free survival and (b) overall survival according to the recurrence pattern at the time of lenvatinib therapy initiation.**

(a)

(b)
